# Radiomics-based biomarker for PD-1 status and prognosis analysis in patients with HCC

**DOI:** 10.3389/fimmu.2025.1435668

**Published:** 2025-01-29

**Authors:** Gulizaina Hapaer, Feng Che, Qing Xu, Qian Li, Ailin Liang, Zhou Wang, Jituome Ziluo, Xin Zhang, Yi Wei, Yuan Yuan, Bin Song

**Affiliations:** ^1^ Department of Radiology, West China Hospital, Sichuan University, Chengdu, Sichuan, China; ^2^ Institute of Clinical Pathology, Key Laboratory of Transplant Engineering and Immunology, West China Hospital, Sichuan University, Chengdu, Sichuan, China; ^3^ Pharmaceutical Diagnostics, General Electric (GE) Healthcare, Shanghai, China; ^4^ Department of Radiology, Sanya People’s Hospital, Sanya, China

**Keywords:** hepatocellular carcinoma, PD-1, radiomics, computed tomography, overall survival

## Abstract

**Purpose:**

To investigate the impact of preoperative contrast-enhanced CT-based radiomics model on PD-1 prediction in hepatocellular carcinoma (HCC) patients.

**Methods:**

The study included 105 HCC patients (training cohort: 72; validation cohort: 33) who underwent preoperative contrast-enhanced CT and received systemic sorafenib treatment after surgery. Radiomics score was built for each patient and was integrated with independent clinic radiologic predictors into the radiomics model using multivariable logistic regression analysis.

**Results:**

Seventeen radiomics features were finally selected to construct the radiomics score. In multivariate analysis, serum creatine and peritumoral enhancement were significant independent factors for PD-1 prediction. The radiomics model integrated radiomics signature with serum creatine and peritumoral enhancement showed good discriminative performance (AUC of 0.897 and 0.794 in the training and validation cohort). Overall survival (OS) was significantly different between the radiomics-predicted PD-1-positive and PD-1-negative groups (OS: 29.66 months, CI:16.03-44.40 vs. 31.04 months, CI: 17.10-44.07, P<0.001). Radiomics-predicted PD-1 was an independent predictor of OS of patients treated with sorafenib after surgery. (Hazard ratio [HR]: 1.61 [1.23-2.1], P<0.001).

**Conclusion:**

The proposed model based on radiomic signature helps to evaluate PD-1 status of HCC patients and may be used for evaluating patients most likely to benefit from sorafenib as a potentially combination therapy regimen with immune checkpoint therapies.

## Introduction

Hepatocellular carcinoma (HCC) is one of the most common hepatic malignant tumors and the third leading cause of cancer-related death worldwide (1). Surgical resection and liver transplantation are potentially curative but are greatly hampered by high recurrence rates (2). The overall outcomes remain unsatisfactory due to its highly heterogeneous malignancy at the genomic, molecular, and histologic levels (3, 4).

Emerging treatments targeting immune checkpoint or multikinases have shown promising efficiency against this devastating disease both in pre-operation and post-operation (5, 6). Immunotherapy via programmed cell death protein 1 (PD-1) and programmed death ligand 1 (PD-L1) checkpoint blockade, reactivating T cell–mediated antitumor immunity by blocking the interaction between PD-1 and PD-L1, have shown encouraging clinical results (7). Anti-PD-1 blockade immunotherapy helps to increase the objective response rates of sorafenib-pretreated patients with HCC to 15%~20%, three times greater than that of the sorafenib-only treatment (5, 8, 9). Despite the remarkable improvement in clinical benefit, PD-1 or PD-L1 blockades so far still benefit only a minority of patients with HCC and the durable response rate to anti-PD-1 therapy remains relatively low, approximately 15%–20%, in patients with HCC (5, 10). A appropriate approach to discriminate patients who can drive utmost benefits from PD-1 blockade therapy is urgently needed. A preoperative noninvasive way to predict PD-1 status may help to guide individualized HCC treatment and better estimate tumor outcome.

Radiomics is a new computerized format that converts medical images into quantitative data. Furthermore, based on the premise that radiomics data can better reflect the microenvironment of tumors, thus, the high-dimensional data provides a deep and comprehensive characterization of tumor heterogeneity, allowing for more personalized and accurate classification of tumor immunophenotypes (11, 12). Previous studies have revealed that MRI-based radiomics features show significantly correlations with HCC immuno-oncological characteristics and potentially with outcome (13). Specifically, the favorable predictive value of radiomics analysis for HCC molecular status have also been reported, Wang et al. have reported the radiomics characteristics of gadoxetic acid–enhanced MRI as biomarkers for predicting cytokeratin19 status in HCCs, separating a subtype of hepatic progenitor cell origin HCCs (14),Gong et al. suggest that a radiomics model based on multisequence MRI has the potential to predict the preoperative expression of PD-1 and PD-L1 in HCC, which could become an imaging biomarker for immune checkpoint inhibitor (ICI)-based treatment (15).However, to our knowledge, no study before has investigated whether the contrast-enhanced CT-based radiomics feature can be used to predict the PD-1 expression status and further illustrate its prognostic value of HCC.

This study aims to determine whether preoperative radiomics based on contrast-enhanced computed tomography (CECT) imaging may help identify the PD-1 positive patients with HCC and further investigate its correlation with prognosis in patients using sorafenib after surgery.

## Materials and methods

### Patient cohort

**Figure 1:**
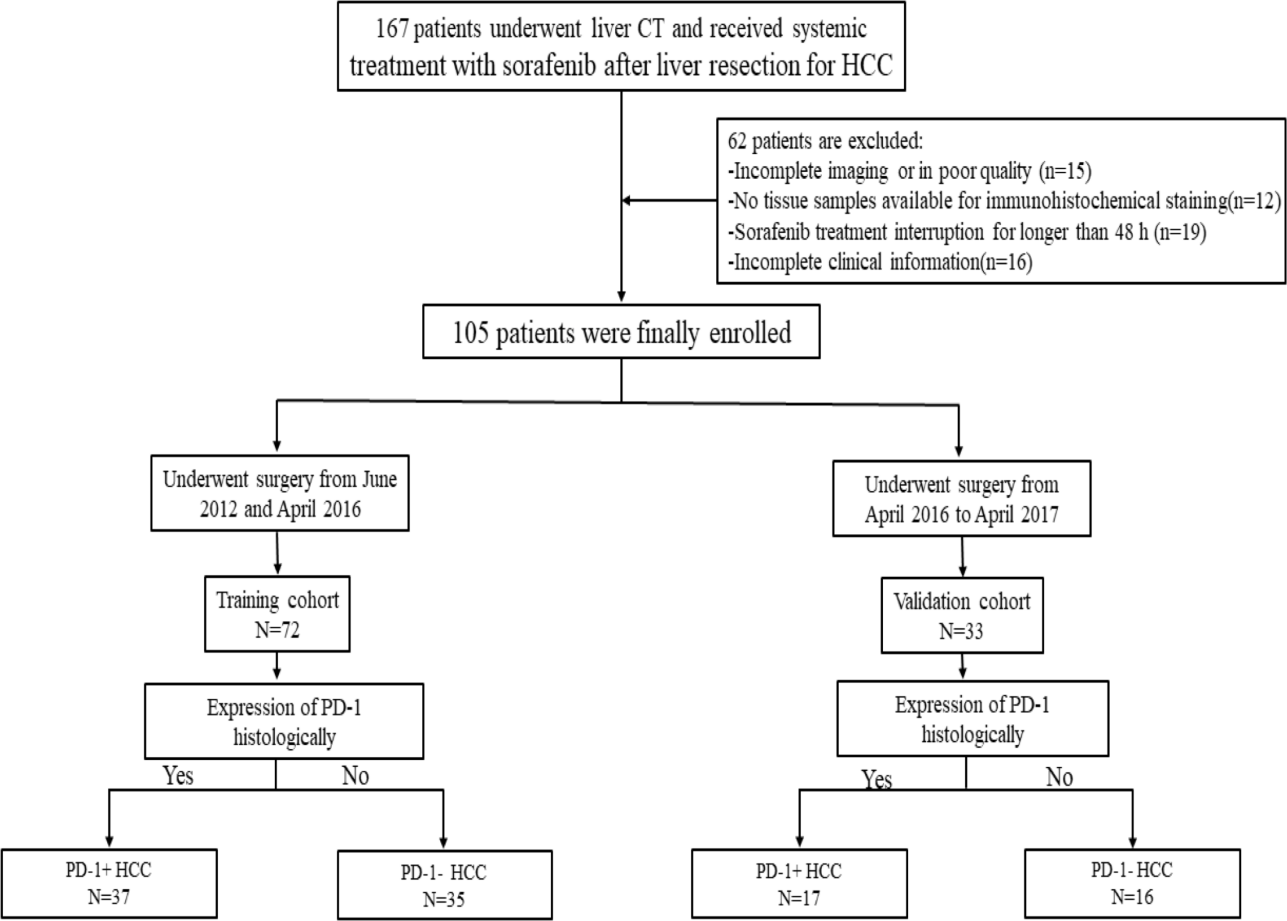
Flow diagram of this study.

This study was approved by the institutional ethical review board of our institution and complied with the Declaration of Helsinki. The informed consent was waived owing to the retrospective study design. The study cohort was collected from our institutional radiology and pathology database between June 2012 and October 2017. A total of 167 patients who had histologically proven HCC and received systemic treatment with sorafenib at standard recommended daily dose of 800 mg (400 mg twice daily) after surgery were included. The inclusion criteria were as follows: (1) age ≥18 years; (2) primary pathologically confirmed HCC; (3) interval between CECT examination and surgery less than four weeks; (4) no local-regional therapy before CECT such as transcatheter arterial chemoembolization (TACE) or radiofrequency ablation (RFA). Then, 62 patients were excluded for the following exclusion criteria: (1) the CT images were incomplete or had poor image quality (n=15); (2) no formalin-fixed, paraffin-embedded tissue samples available for immunohistochemical staining(n=12); (3) incomplete clinical information(n=16); and (4) sorafenib treatment was interrupted for longer than 48 hours between the initiation of sorafenib and the first follow-up time point (n=19). Therefore, 105 patients were finally enrolled in this study and patients who underwent surgery between June 2012 and April 2016 constituted the training cohort(n=72), and the subsequent patients who underwent surgery from May 2016 to October 2017 constituted the validation cohort(n=33) (**Figure 1**). Data on demographics, laboratory tests, tumor pathology and clinical conditions were derived from the patients’ medical records.

### Patient follow-up

After resection, patients were followed up and was screened by means of serum a-fetoprotein level, liver function tests, and contrast-enhanced CT or MRI of the chest and abdomen every 3 months during the first 2 years and then every 6 months thereafter. The data were censored on December 15, 2021. Overall survival (OS) was measured as the interval from the date of surgery to the date of death from a disease-related cause or the latest follow-up.

### Immunohistochemistry staining

The paraffin tissue from surgically resected specimens were cut into 4 μm-thick sections, dewaxed, hydrated, and then antigen retrieval. Then, tissue slides were incubated with primary antibodies using rabbit anti-human PD-1 polyclonal antibody (5 μg/ml, cat # PA5-20351; Invitrogen; Thermo Fisher Scientific, Inc., Waltham, MA, USA.) at 4°C overnight, followed by incubation with secondary antibody (cat # K5007; Dako). PD-1 staining was performed with 3,3’-diaminobenzidine and counterstained with hematoxylin. Two senior pathologists independently selected five non-overlapping and discontinuous regions to calculate the mean for statistical analysis. The numbers of PD-1 cells were quantified at ×400 (0.0484 mm2) and the cut-off values for PD-1 overexpression were determined by x-tile software based on the current study. Cases with expression greater than 5% were considered as PD-1-positive (16, 17).

### CT examination

The CT images was obtained in the multidetector CT scanners (Revolution, GE Healthcare, Milwaukee, USA; SOMATOM definition, Siemens Healthcare, Erlangen, Germany). Triple-phase CT examinations were conducted, i.e., non-enhanced, arterial, and portal vein phases. The arterial phase and portal venous phase were obtained at 25-30 s and 60-70 s after contrast injection. The detailed scanning parameters are listed as follows: tube voltage, 100-120 kVp; tube current, 450 mA; slice thickness, 0.625 mm; pitch, 0.992:1; rotation speed: 0.5 s/rot; and ASIR-V: 30%. All patients received an intravenous, non-ionic contrast agent (iodine concentration, 300-370 mg/mL; volume, 1.5–2.0 ml/kg of body weight; contrast type, iopromide injection, Bayer Pharma AG) injection.

### CT analysis

Two board-certified radiologists (reader 1 and reader 2 with 17 and 8 years of experience in abdominal imaging, respectively), who were blinded to the clinicopathology and follow-up results, independently reviewed all CT images. The following imaging features were evaluated: 1) tumor size was measured as the maximum tumor diameter on the axial CT image; 2) ill border, defined as non-smooth margin with budding portion protruding into the liver parenchyma or infiltrative appearance at the tumor periphery, otherwise as smooth margin; 3) pseudo-capsule, defined as complete capsule when observing a uniform border around most or all of the tumor, unequivocally thicker or more conspicuous than fibrotic tissue around background nodules, otherwise as incomplete integrity or not applicable; 4) multifocality; 5) arterial phase(AP) hyperenhancement; 6) portal venous phase (PVP)hypoenhancement; 7) radiologic evidence of necrosis; 8) peritumoral enhancement was defined as a detectable portion enhanced in the arterial phase adjacent to the tumor border, later turning isoattenuation in the equilibrium phase; and 9) portal vein tumor thrombosis invasion. After the first independent image analysis, interobserver agreement for the assessment of the CT imaging features was evaluated. The two reviewers then met to discuss final conclusions by consensus on discordant results. All examinations were performed using a workstation and recorded on a picture archiving and communication system (Syngo-Imaging, version VB36A; Siemens Medical Solutions).

### Radiomic feature extraction and selection

Using ITK-SNAP software (version 3.6.0), a region of interest (ROI) was manually drawn around the contour of the tumor slice to exclude tumor necrosis and calcification by reader3 and reader4 (with 5 and 6 years of experience in abdominal imaging, respectively). Radiomic features were generated from the images using Internal Scientific 3D Analysis software (Analysis Suite, version V3.0.0). R, GE Healthcare). Two resource extraction methods are extracted. Raw feature class and 14 filtered classes (boxmean, additivegaussiannoise, binomialblurimage, curvatureflow, boxsigmaimage, log, wavelet, normalize, laplaciansharpening, discretegaussian, mean, specklenoise, recursivegaussian and shotnoise). A total of 2,600 radioactive elements were identified for further analysis. The training suite feature selection process consists of three steps: Firstly, variance analysis. Secondly, we performed Spearman’s correlation test and features with the coefficients greater than 0.95 were excluded due to the redundancy. Then, we applied LASSO method with a 10-fold cross validation applied to select the most powerful features in the training set. And the features were standardized with replacing missing values by median and Z-score normalization.

The established multivariate logistic regression model was used to calculate the radiomics score for each patient. Reader 3 and reader 4 repeated the feature extraction twice during a 1-week period to evaluate the intra-observer reliability. The inter-observer reliability and intra-observer reliability were assessed by obtaining the intraclass correlation coefficient (ICC). Features with ICC values >0.75 were selected for subsequent investigation.

### Model development and validation

The radscore was computed for each patient by a linear combination of selected features weighted by their respective coefficients. Clinical-radiological characteristics and radiomics features significantly associated with PD-1 in the univariate analyses were included in the multivariate logistic analysis to identify significant predictors based on a backward stepwise selection process with the Akaike information criterion. The radiomics model was formulated based on the results of multivariate regression. Correspondingly, one clinical model was generated on the basis of semantic features and clinical parameters. The radiomics model is based on proportionally converting each regression coefficient in multivariate logistic regression to a 0- to 100-point scale. The effect of the variable with the highest b coefficient (absolute value) is assigned 100 points. The points are added across independent variables to derive total points, which are converted to predicted probabilities (Pi).

### Statistical analysis

Continuous and categorical variables were compared using t-test and the chi-square test, respectively. After analyzing potential factors in univariate logistic regression analysis, a multivariable regression analysis was performed to select the independent risk factors of PD-1 expression. The discrimination performance of the prediction models was quantified by the area under the curve (AUC) of receiver operator characteristic curve (ROC) value in the training and validation cohorts. Model fitness was assessed by calibration curves with the Hosmer-Lemeshow test. Additionally, the decision curve analysis was performed to evaluate the clinical usefulness and net benefits of the developed prediction models, which estimates the net benefit of a model as the difference between the true-positive and false-positive rates, weighted by the odds of the selected threshold probability of risk. Survival curves of OS were generated according to the Kaplan-Meier method and compared using the log-rank test. Univariate analyses with Cox proportional hazards regression determined the predictors of mortality. The inter-observer agreement was applied to assess the reliability of imaging analysis using the Kappa test; 0–0.2 represents slight, 0.21–0.40: fair, 0.41–0.60: moderate, 0.61–0.80: substantial, 0.81–1: excellent.

Statistical analysis was conducted with R software (version 3.4.2, http://www.R-project.org) and SPSS software (version 22.0, IBM). A two-sided P values < 0.05 was indicative of a statistically significant difference.

## Results

### Patient characteristics

**Figure 2:**
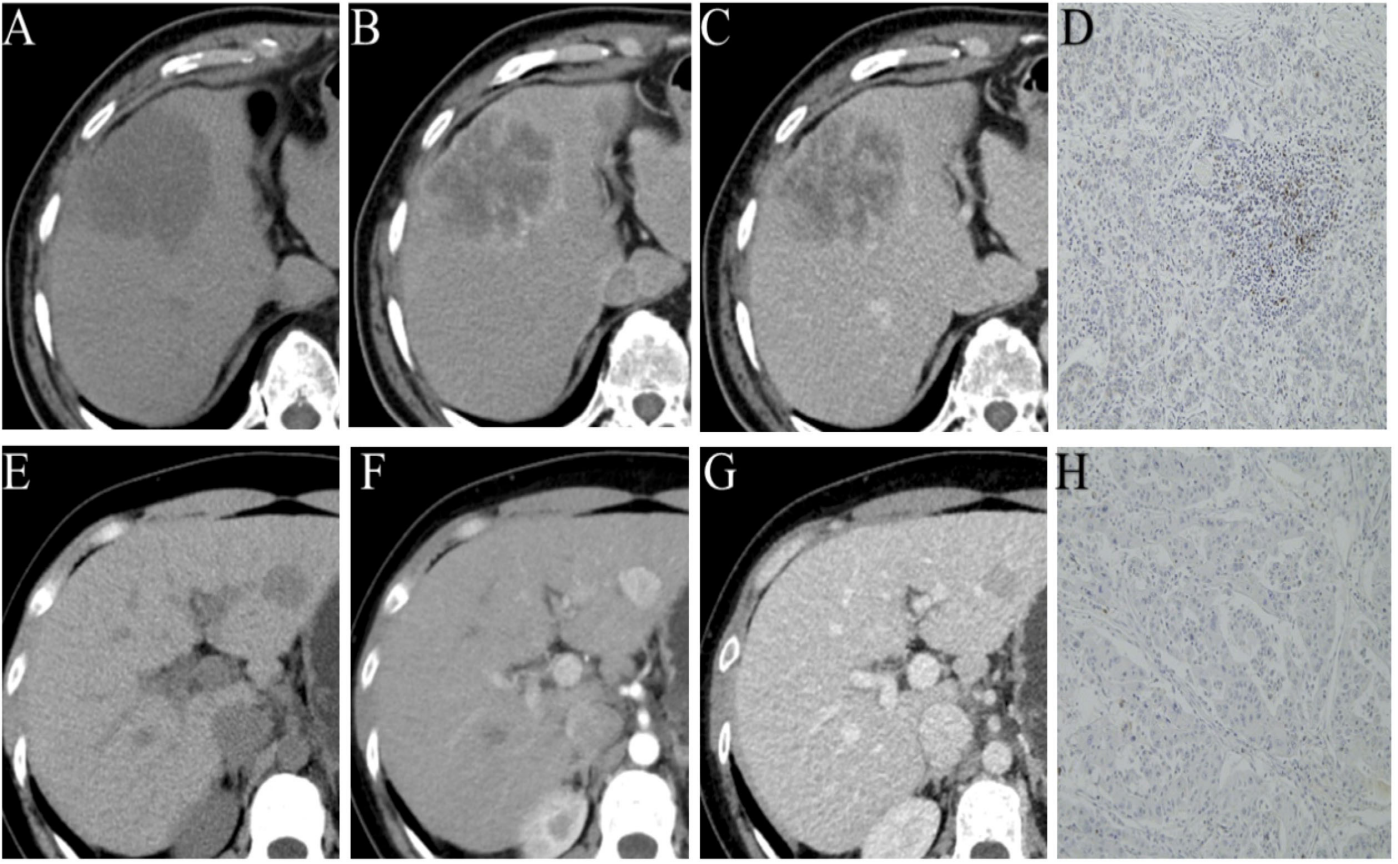
Representative images of different phases of CECT and PD-1 status by immunohistochemical staining (PD-1 positive **(A–D)**; PD-1 negative **(E–H)**.

The characteristics of the patients are summarized in **Table 1**. Out of 105 patients included (male/female: 92/13; mean age, 51.17 ± 12.23 years, range 21 to 78 years), PD-1 was identified in 51 (48.6%) patients (**Figure 2**). According to the Kaplan-Meier analysis, PD-1-negative patients lived longer than PD-1-positive patients (OS: 25.57 months; 95% confidence interval [CI]:15.75-41.84 vs. 31.23 months; CI: 16.51-45.42; P<0.05).

**Table 1:** Baseline characteristics of patients in the training and validation cohorts.

Baseline variables	Training cohort (n=72)		*P* value	Validation cohort (n=33)		*P* value
	PD-1-positive (37)	PD-1-negative (35)		PD-1-positive (17)	PD-1-negative (16)	
Gender (Male, %)	33 (89.2)	29 (82.9)	0.663	16 (94.11)	14 (87.5)	0.601
Age (mean ± SD,years)	53.46 ± 11.80	51.09 ± 11.67	0.394	50.88 ± 11.70	46.38 ± 14.52	0.332
HBV (+; %)	33 (89.19)	32 (91.43)	0.938	16 (94.12)	14 (87.5)	0.601
ALT (>40IU/L, %)	6 (16.22)	7 (20)	0.677	4 (23.53)	1 (6.25)	0.335
AST (>35IU/L, %)	27 (73.0)	24 (68.6)	0.681	6 (35.29)	8 (50.)	0.491
AFP (>400ng/ml, %)	24 (64.86)	23 (65.71)	0.94	8 (47.06)	12 (75.)	
CEA (>3.4ng/ml, %)	2 (5.41)	4 (11.43)	0.619	3 (17.65)	1 (6.25)	0.601
TBIL (umol/L; IQR)	15 (11.79, 20.05)	17.2 (11.3, 23.16)	0.834	14.8 (10.67, 19.41)	18.2 (10.48, 22.63)	0.449
PLT (×10^9/L; IQR)	149 (113.4, 210.2)	135 (84, 166.4)	0.152	144.5 (120.7, 211.3)	154 (111.95, 186.7)	0.943
Creatinine (u/L; IQR)	80 (67.8, 88.9)	67 (59, 78.2)	0.004	76.9 (65.91, 89.3)	69.5 (61.8, 81.65)	0.214
ALB (g/L; IQR)	43.2 (38.11, 46)	43.7 (40.94, 47.2)	0.201	44.2 (39.73, 47.56)	44 (41.47, 47.02)	0.692
GGT (u/L; IQR)	50 (33.1, 102.3)	60 (26, 101)	0.991	75 (50, 138.3)	77 (40.05, 147.9)	0.857
MVI (+; %)	3 (8.11)	1 (2.86)	0.647	8 (47.06)	9 (56.25)	0.732
Differentiation (poor, %)	21 (56.76)	23 (65.71)	0.436	6 (35.29)	10 (62.5)	0.169
BCLC (%)						
0-A	4 (10.8)	10 (28.6)	0.324	1 (5.9)	4 (25.0)	0.313
B	22 (59.5)	15 (42.9)		9 (52.9)	7 (43.8)	
C	11 (29.73)	10 (28.6)		7 (41.2)	5 (31.3)	
Imaging findings						
Tumour size (>5cm; %)	19 (51.35)	19 (54.29)	0.803	10 (58.82)	11 (68.75)	0.721
Multifocality (%)	11 (29.73)	10 (28.57)	0.914	4 (23.5)	1 (6.3)	0.335
Ill border (%)	16 (43.2)	17 (48.6)	0.65	9 (52.9)	10 (62.5)	0.728
Portal invasion (%)	18 (48.7)	12 (34.3)	0.217	8 (47.1)	9 (56.3)	0.732
Pseudo–capsule (%)	19 (51.4)	14 (40.0)	0.334	9 (52.9)	7 (43.8)	0.732
AP hyperenhancement (%)	34 (91.9)	33 (94.3)	0.949	14 (82.35)	15 (93.8)	0.601
PVP hypoenhancement (%)	34 (91.9)	33 (94.3)	0.949	16 (94.11)	14 (87.5)	0.601
Radiologic evidence of necrosis (%)	27 (77)	23 (65.7)	0.504	10 (58.8)	11 (68.8)	0.721
Peritumoral enhancement (%)	18 (48.7)	12 (34.3)	0.217	7 (41.2)	9 (56.3)	0.494

Unless otherwise indicated, data are the number of patients. HBV, hepatitis B surface antigen; ALT, alanine aminotransferase; AST, aspartate aminotransferase; AFP, alpha-fetoprotein; CEA, carcinoembryonic antigen; TBIL, total bilirubin; PLT, platelet count; ALB, albumin; GGT, γ-glutamyl transpeptidase; MVI, microvascular invasion; BCLC, Barcelona Clinic Liver Cancer; SD, standard deviation; AP, arterial phase; PVP, portal venous phase.

**Figure 3:**
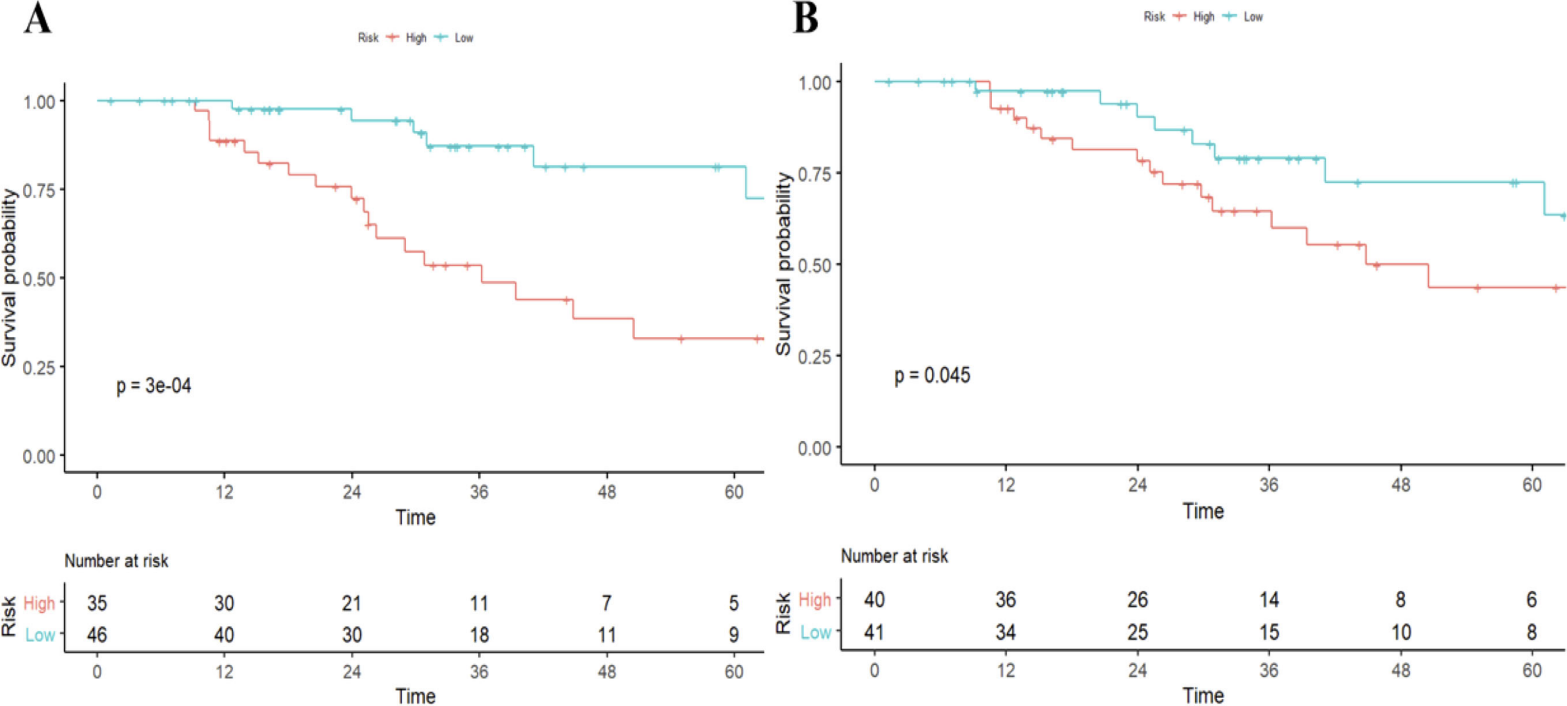
Survival curves according to histological and radiomics model-predicted PD-1 status. OS curves scaled by histologic PD-1status **(A)** and radiomics model-predicted PD-1 status **(B)** with Kaplan-Meier analysis. The classification of radiomics model-predicted PD-1 status was derived using the optimal threshold that maximizes the Youden index of receiver-operating characteristic analysis. OS, overall survival.

As of December 2021, 92/105 (87.7%) had completed the OS follow-up, of which 81 patients 77.1%) died of progression of HCC and 11 patients died of other cause (10.5%). Univariable Cox regression analysis showed that histologic PD-1 (hazard ratio [HR], 4.1; 95% CI: 1.79,9.39; P<0.001) and the radiomics model Pi (HR, 1.61; 95% CI: 1.23-2.1; P<0.001) were independent predictors of disease-specific mortality. The median OS was 25.57(15.75-41.84) months for those with PD-1 positive, and 31.23 (16.51-45.42) months for those with PD-1 negative (log-rank test,P<0.05.) The median OS was 29.66 (16.03-44.40) months for those with RR-predicted PD-1 negative and 31.04 (17.10-44.07) months for those with RR-predicted PD-1 positive (log-rank test, P<0.001) (**Figure 3**).

### Radscore construction

Among 2600 radiomic features, 358 features with high correlation were identified for univariable logistic regression analysis. Variables with P<0.05 in the univariable logistic regression analysis were included in the multivariable regression model with backward stepwise selection using the Akaike information criterion. Eventually, 17 radiomic features related to PD-1 expression were selected to construct radscore (**Supplementary Data Sheet 1**). The radiomics signature indicated favorable prediction of PD-1 status with an AUC of 0.852 (95% confidence interval [CI]: 0.76, 0.94) in the training cohort and 0.79 (95% CI: 0.63-0.95) in the validation cohort.

### Prediction model for identifying PD-1

Univariable logistic regression analysis showed that radscore (odds ratio (OR), 3.412; 95% confidence interval (CI), 1.562 to 7.453, P = 0.002), ALT level (OR, 0.159; 95% CI, 0.038 to 0.673, P < 0.012), tumor size (OR, 0.243; 95% CI, 0.059 to 1.003, P = 0.05) and tumor margin (OR, 0.170; 95% CI, 0.044 to 0.664, P = 0.011) were independently vulnerable to histologic PD-1, and these significant factors were then selected into a multivariate logistic regression, and at the multivariate analysis, serum creatinine (p = 0.063, OR 0.95, 95% CI 0.91–1), peritumoral enhancement (p = 0.091, OR 3.12, 95% CI 0.83–11.7), and radiomics scores (p <0.001, OR 2.63, 95%CI 1.54–4.47) were all independent significant variables associated with PD-1 expression (**Table 2**). The Akaike information criterion (AIC) was calculated to assess the risk of data overfitting.

**Table 2:** Univariate and multivariate analyses of factors related with PD-1 status.

Risk factors	Univariate analysis		Multivariate analysis	
	OR (95% CI)	*P* value	OR (95% CI)	*P* value
Creatinine	0.95 (0.92-0.99)	0.007^*^	0.95 (0.91-1)	0.063
Peritumoral enhancement	2.77 (1.07-7.20)	0.037^*^	3.12 (0.83-11.7)	0.091
Radiomics Scores	2.72 (1.63-4.53)	<0.001^*^	2.63 (1.54-4.47)	<0.001^*^

* referred to P<0.05; CI, confidence interval.

**Figure 4:**
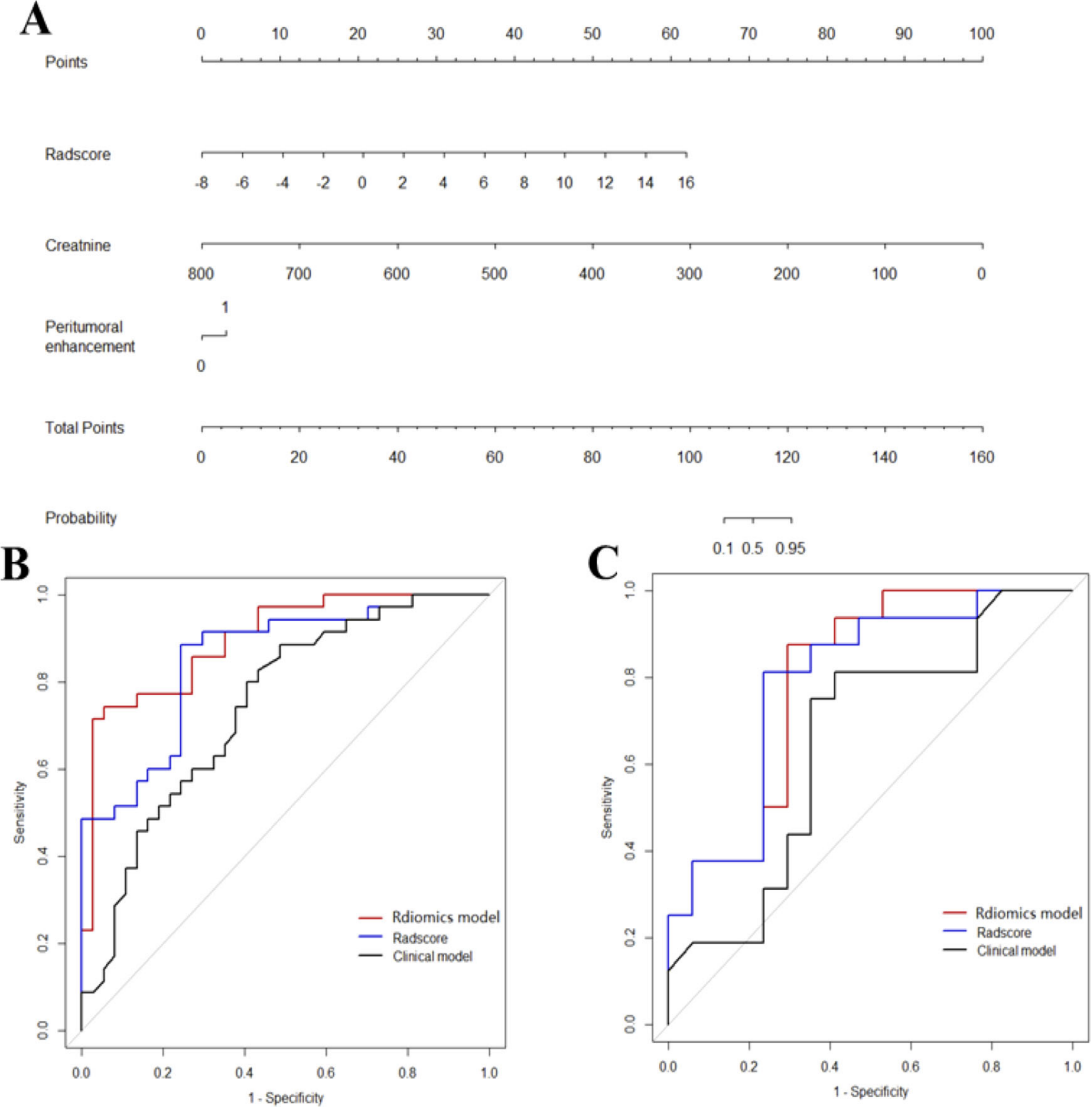
Diagnostic performance of the three models. The developed radiomics model nomogram **(A)** for PD-1 status prediction, which is scaled by the proportional regression coefficient of each predictor. Comparison of receiver operating characteristic curves of the radscores, clinical model and radiomics model in the training **(B)** and validation **(C)** cohorts.


Incorporated with these independent risk factors, the radiomics model yielded a good performance for stratifying PD-1 status and presented as a nomogram to provide individualized risk estimates (**Figure 4**). The formula of the radiomics model for predicting PD-1 is given below:

$$\begin{array}{c} \text{Y }(\text{PD}-1)=0.965*\text{Radscore}-0.047*\text{Creatinine}\\ +1.139*\text{Peritumoral enhancement}+2.918 \end{array}$$

Accordingly, a clinical model was produced with moderate performance. The formula of the clinic radiologic model for predicting PD-1 is given below:


$$\begin{array}{c} \text{Y }(\text{PD}-1)=-0.0432*\text{Creatinine}\\ +0.923*\text{Peritumoral enhancement}+2.679 \end{array}$$

Excellent interobserver agreement was observed for the imaging feature evaluation, with Kappa value of 0.830 for peritumoral enhancement.

### Model evaluation

**Figure 5:**
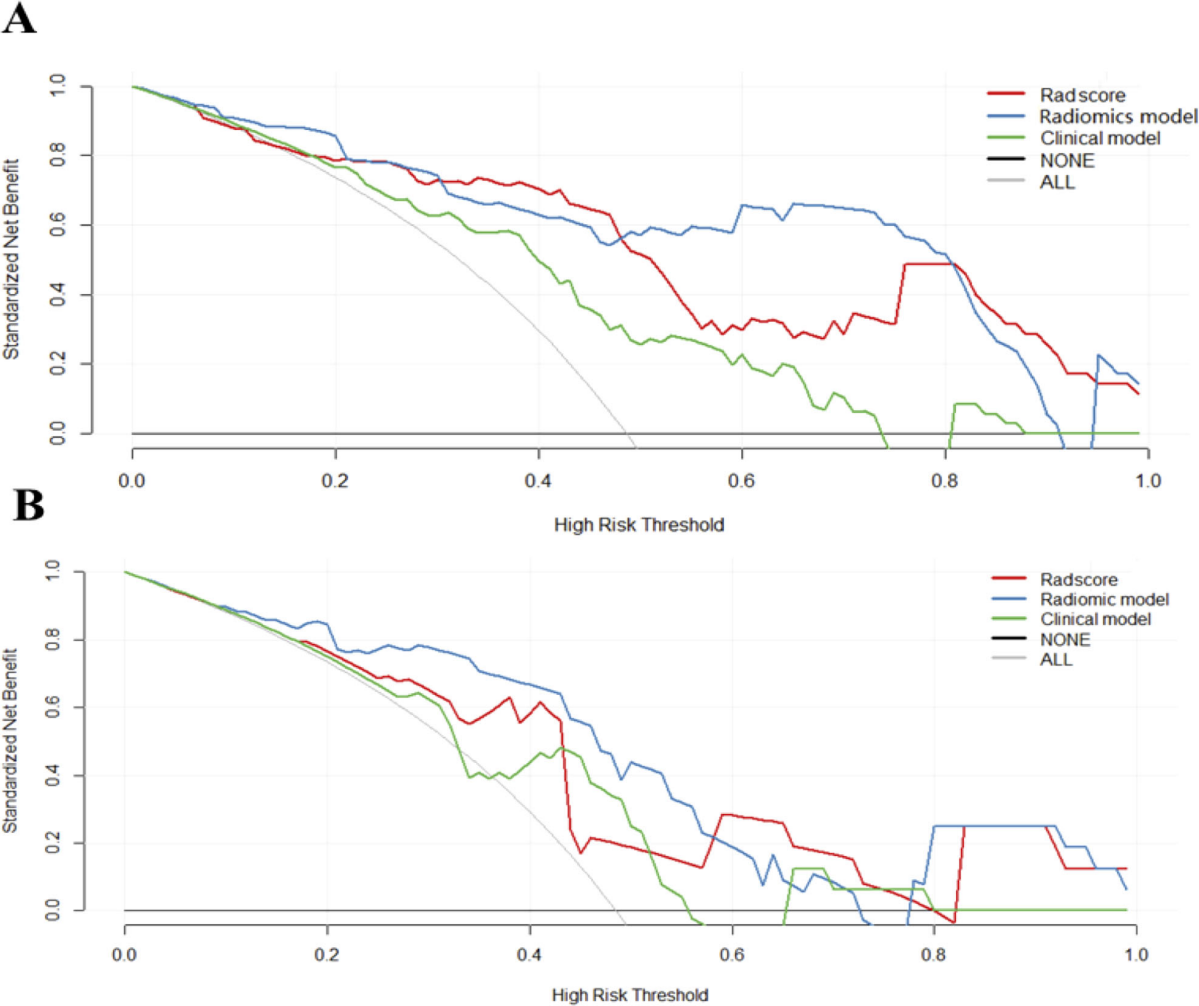
Decision curves of each model. Decision curves analysis of three models in the training **(A)** and validation cohorts **(B)**. The y-axis measures the net benefit, which was calculated by summing the benefits (true-positive results) and subtracting the harms (false-positive results). The radiomics model provided the highest net benefit compared with the radscores and clinical model.

ROC curves of validation group showed stability of the radiomics model (**Figure 3**). The diagnostic performance of the radiomics model was superior to that of clinical model with AUCs of 0.897 (95% CI, 0.83- 0.97) vs 0.742 (95% CI, 0.627-0.856) in training cohort and 0.794 (95% CI,0.636-0.952) vs 0.651 (95% CI, 0.454-0.847) in validation cohort, with a sensitivity of 88.6%, specificity of 75.7% in training cohort and sensitivity of 87.5%, specificity of 52.9% in validation cohort (**Table 3**). Radiomicd model show good specificity, the clinical model and radiomics score showed better sensitivity relatively. The calibration curve analysis demonstrates that PD-1 status predicted by the radiomics model fitted well with the actual PD-1 phenotypes in both cohorts. As part of this study, we also tested the incremental value of the radscores with respect to clinical model for predicting PD-1 using a decision curve analysis, as described in **Figure 5**. Decision curves analysis demonstrated that the radiomics model combined with clinical model and radscores did provide a net benefit compared with the clinical model.

**Table 3:** Performance of prediction model in the training and validation cohorts.

Model	Training group			*P* value	Validation group			*P* value
	AUC (95% CI)	Sensitivity	Specificity		AUC (95% CI)	Sensitivity	Specificity	
Radiomics score	0.852 (0.764-0.940)	88.6%	75.7%	0.137^1^	0.79 (0.630-0.951)	87.5%	52.9%	0.284^1^
Clinical model	0.742 (0.627-0.856)	88.6%	51.4%	0.002^2^	0.651 (0.454-0.847)	81.2%	47.1%	0.226^2^
Radiomics model	0.897 (0.825-0.968)	74.3%	94.6%	0.441^3^	0.794 (0.636-0.952)	75.0%	70.6%	0.905^3^

^1^AUCs of radiomics score and clinical model were compared; ^2^AUCs of clinical model and radiomics model were compared; ^3^AUCs of radiomics score and radiomics model were compare.

## Discussion

In this study, we established and validated a radiomics model based on CECT images for PD-1 prediction in patients with HCC. We concluded that radiomic features are of great value to complement clinicopathologic features for PD-1 prediction. In addition, the PD-1 status predicted by the model were independently associated with long-term mortality which suggests that our model could potentially be used for evaluating patients most likely to benefit from sorafenib as a potentially combination therapy regimen with immune checkpoint therapies.

Current acquisition of tumor’s immune microenvironment is based on histological specimens which is limited by intratumoral heterogeneity comparted with whole tumor analysis of radiomics. In this study, radiomics features provided increased power (AUC = 0.852) for PD-1 prediction in HCC patients and were indicated to be independent predictors for PD-1 in the final radiomics model. The radiomics model used in our study significantly outperformed the clinical model in discriminatory ability (AUC 0.897 vs 0.752 in training cohort; AUC 0.794 vs 0.651 in validation cohort). Previous studies have used radiomics analysis for noninvasive assessment of molecular and clinical-pathologic characteristics of tumors (18–20). Wang Q et al. proposed an RF-based radiomics analysis method for PD-1 prediction in HCC patients (21). Yuan G et al. developed a radiomics nomogram at patient-level based on CECT to predict the anti-PD-1 treatment efficacy in patients with advanced HCC (22). Both a lesion-based approach and patients-based approach were applied in the study of Cui H et al. to evaluate the response of anti-PD-1 therapy in patients with HCC based on CECT radiomics model (23). These previous studies whether used radiomics analysis based on ultrasound multi-feature maps for PD-1 expression prediction or CECT radiomics method to predict the therapeutic efficacy of PD-1 immunotherapy. No study before tries to combine radiomics analysis based on CECT images with PD-1 prediction in HCC patients. In this study, we developed a satisfactory radiomics model. Among the features selected for the fusion Rad-score construction, the wavelet-based features occupied more than half (10/17) in our study, which shows the vital role of wavelet-based features in the prediction model for they can better explore the spatial heterogeneity of tumors at multiple scales (24, 25).

During the preoperative work-up, the clinicoradiologic features of serum creatinine levels (P =0.007) and the peritumoral enhancement (P =0.037) were independently associated indicators for preoperative evaluation of PD-1 in HCC patients. Previous studies have showed that serum creatinine can be independent predictors for hepatic decompensation (26). Lambrecht J et al. developed a novel blood-based diagnostic APAC score, consisting of age, soluble platelet-derived growth factor receptor beta, AFP and creatinine, for early diagnosis of HCC patients with liver cirrhosis (27). Chen Y et al. to study the risk factors of AKI after liver transplantation, and the results show that chronic severe hepatitis and preoperative creatinine may be potential risk factors for the occurrence of AKI after liver transplantation (28). Ho CT et al. developed a risk scores using conventional methods and ML(machine learning) to categorize early-stage HCC patients into distinct prognostic groups. Factors for the CATS-IF score were selected by the conventional method, including age, curative treatment, single large HCC, serum creatinine and alpha-fetoprotein levels, fibrosis-4 score, lymphocyte-tomonocyte ratio, and albumin-bilirubin grade, and both the conventional Cox-based CATS-IF score and ML-based CATS-INF score effectively stratified patients with early-stage HCC into distinct prognostic groups (29). Our final radiomics model incorporates creatinine as a predictive feature suggesting a relationship between creatinine and PD-1 status, in accordance with previous results of creatinine prediction function for liver cancer (27, 30). The peritumoral enhancement was shown to be associated with PD-1 expression. It may be related to peritumoral hemodynamic changes caused by positive PD-1. PD-1, known to be a key immune-checkpoint receptor, functions primarily in peripheral tissues where T cells may encounter the immunosuppressive PD-L1 and PD-L2. By blocking effector functions between PD-1 and PD-L1 and reducing T-cell killing capacity, PD-1 positive cells tend to grow with invasive margin with more angiogenesis.

The multi-kinase inhibitor sorafenib, which is the only approved agent recommended by the AASLD for advanced HCC, is currently recommended as the first-line therapy in these patients. STORM trial show the median DFS in the sorafenib group was not significantly improved compared with the placebo group (31). The network meta-analysis (32) show sorafenib had the trend of increasing recurrence rate, but there were no significant differences between them, however some subsequent studies have made different findings (33–35) whether sorafenib should be used exclusively as adjuvant therapy for HCC after curative resection remains controversial, this would need to be confirmed by more regional, well-designed randomized controlled trials (36). A unified guide is still lacking today, In clinical practice, comprehensive analysis is needed, according to the specific situation of patient choose the appropriate adjuvant therapy methods.

Decrease in PD-1 expression on T cells has been reported to be associated with improved HCC prognosis in patients following sorafenib treatment, prior anti-PD-1 antibody treatment can amplify HCC response to sorafenib therapy (37, 38). Greater decreases in the numbers of CD4+PD-1+ T cells or CD8+PD-1+ T cells after sorafenib therapy were correlated with better OS, which indicates the immunomodulatory effect of sorafenib.

Many scholars have applied radiomics for postoperative prognosis prediction of HCC (39). Yu et al. reported that the intratumoral or peritumoral radiomics model could predict prognosis for HCC patients with vessels encapsulating tumor clusters (40). In our study, PD-1 was associated with OS and the radiomics-predicted PD-1 was an independent predictor of OS in patients using sorafenib treatment after surgery. These suggest that our computational-assisted model could potentially be used for evaluating patients most likely to benefit from sorafenib as a potentially combination therapy regimen with immune checkpoint therapies, enhancing the immune-based strategies therapeutic efficacy against advanced malignancies.

Our study had several limitations. Firstly, our study was developed on a limited sample size collected retrospectively in a single center, additional external cohorts with a large study population are needed for its stability and generalizability. Secondly, because specimen staining is required, a large number of patients with advanced HCC who are losing chance for surgery because of bad condition were excluded. Potential selection bias may influence the OS results, since sorafenib treatment is more commonly used in that kind of patients. Thirdly, multi-parametric MRI has assumed to provide muti-scale information of liver neoplasia, especially MRI with gadoxetic acid, could provide more efficient information is worthy of exploration.

## Conclusion

In conclusion, the proposed combined model based on CT radiomic signatures demonstrate good performance for preoperatively prediction of PD-1 in HCC patients. The radiomics features may provide a promising opportunity to improve clinical decision support for patients with immunotherapeutic approaches. Although our study showed a high predictive power, future research with external independent cohorts and prospective validations were required to validate it before translating into clinical implementation.

## Data Availability

The raw data supporting the conclusions of this article will be made available by the authors, without undue reservation.
