# Supplementary material for: Radiomics-based biomarker for PD-1 status and prognosis analysis in patients with HCC
Source: Front Immunol. 2025 Jan 29;16:1435668. doi: 10.3389/fimmu.2025.1435668 (PMC11813882; doi:10.3389/fimmu.2025.1435668)
Supplement: Supplementary file 1 [file DataSheet1.docx]

Radscore=-1.209*wavelet_glcm_wavelet.LLH.Idmn

+0.474*wavelet_glcm_wavelet.HHH.Correlation

+0.775*log_gldm_log.sigma.1.5.mm.3D.DependenceVariance

+0.896*wavelet_glszm_wavelet.LHH.LargeAreaLowGrayLevelEmphasis

+0.753*wavelet_firstorder_wavelet.HHH.Skewness

+0.696*wavelet_glrlm_wavelet.HHL.LongRunHighGrayLevelEmphasis

+5.116*laplaciansharpening_glszm_LargeAreaHighGrayLevelEmphasis

+0.741*discretegaussian_firstorder_Skewness

+-1.209*wavelet_firstorder_wavelet.LLH.Skewness

+0.474*curvatureflow_glszm_SmallAreaLowGrayLevelEmphasis

+0.775*wavelet_gldm_wavelet.LHH.LargeDependenceLowGrayLevelEmphasis

+0.896*log_firstorder_log.sigma.0.5.mm.3D.Mean

+0.753*shotnoise_glrlm_RunVariance

+0.696*normalize_glszm_LargeAreaLowGrayLevelEmphasis

+5.116*wavelet_glszm_wavelet.LHL.SmallAreaLowGrayLevelEmphasis

+0.741*wavelet_firstorder_wavelet.LLH.10Percentile

+-1.209*wavelet_gldm_wavelet.HHL.SmallDependenceLowGrayLevelEmphasis

+ 0.868
